# Handgrip strength, cardiometabolic risk and body composition in youth with type 1 diabetes: the Diactive-1 Cohort Study

**DOI:** 10.1136/bmjsem-2024-002177

**Published:** 2024-12-04

**Authors:** Nidia Huerta-Uribe, Ignacio Hormazábal-Aguayo, Jacinto Muñoz-Pardeza, María J Chueca-Guindulain, Sara Berrade-Zubiri, Carlos Andrés Sesma, Elisabet Burillo Sánchez, Yasmin Ezzatvar, Rodrigo Yáñez-Sepúlveda, Mikel Izquierdo, Antonio García-Hermoso

**Affiliations:** 1 Navarrabiomed, Hospital Universitario de Navarra, IdiSNA, Universidad Pública de Navarra, Pamplona, Spain; 2 Pediatric Endocrinology Unit, Department of Pediatrics, IdiSNA, Hospital Universitario de Navarra, Pamplona, Spain; 3 Lifestyle Factors with Impact on Ageing and Overall Health (LAH) Research Group, Nursing Department, Universitat de València, Valencia, Spain; 4 Faculty of Education and Social Sciences, Universidad Andrés Bello, Viña del Mar, Chile

**Keywords:** Physical fitness, Muscle, Diabetes, Children's health and exercise

## Abstract

**Objective:**

This study aimed to explore the association between handgrip strength, cardiometabolic risk (CMR) and body composition in youth with type 1 diabetes.

**Methods:**

For this prospective cohort study, muscular fitness was assessed via handgrip test and relativised by weight, and body composition, evaluated through dual-energy X-ray absorptiometry in type 1 diabetes patients aged 6–18 years. CMR score included z-scores for total body fat, blood pressure, glycated haemoglobin (HbA1c), low-density lipoprotein cholesterol, high-density lipoprotein cholesterol and triglyceride-glucose index.

**Results:**

Eighty-three patients were analysed at baseline and 1-year follow-up (44.6% females, mean age 12.77 years). Individuals with high handgrip strength tended to have lower CMR and body fat compared with those with low handgrip strength. Over a year, individuals with high handgrip strength showed reduced HbA1c, CMR and subcutaneous fat. Consistently meeting high handgrip strength criteria resulted in reductions in HbA1c levels, CMR score and subcutaneous adipose tissue compared with those who never complied or lost compliance during follow-up. Additionally, subjects classified with high handgrip strength both at baseline and follow-up had a lower likelihood of being classified with high CMR (OR=0.241, 95% CI 0.121 to 0.947, p=0.044).

**Conclusions:**

High handgrip strength was associated with significant cardiometabolic and body composition benefits in youth with type 1 diabetes. This tool could be considered of potential clinical value for incorporating assessments like handgrip tests to monitor and address cardiometabolic health.

**WHAT IS ALREADY KNOWN ON THIS TOPIC**

**WHAT THIS STUDY ADDS**

- High handgrip strength offers significant long-term benefits for cardiometabolic health and body composition in youth with type 1 diabetes.

**HOW THIS STUDY MIGHT AFFECT RESEARCH, PRACTICE OR POLICY**

- Incorporating handgrip tests into routine assessments could provide valuable insights for monitoring and intervening in the cardiometabolic health of youth with type 1 diabetes.

## Introduction

 Cardiovascular disease (CVD) remains the leading morbidity and mortality cause in patients with type 1 diabetes.^1^ Children with type 1 diabetes demonstrate a higher cardiovascular risk factor prevalence versus the general paediatric population,^2^ with up to 45% experiencing two or more risk factors.^3^ Thus, early disease markers may already emerge in this age group, necessitating prompt recognition and management.^4^

The American Diabetes Association identifies physical activity (PA) as integral for improving body composition and preventing CVD in patients with type 1 diabetes, including youth.^5^ Therefore, it is recommended that all youth with type 1 diabetes engage in 60 min of moderate-vigorous daily aerobic activity, supplemented with muscle and bone strengthening exercises at least 3 days per week. Overall, exercise is associated with favourable cardiometabolic changes,^6^ body composition^7^ and psychological outcomes^7^ in children/adolescents with type 1 diabetes. However, resistance training remains less extensively studied despite promising results.^8^

Robust evidence demonstrates the benefits of muscular fitness in youth without type 1 diabetes. However, it is well known that type 1 diabetes significantly impacts the musculoskeletal system and body composition in children and adolescents, often resulting in lower muscle function and higher fat mass compared with their healthy peers. For example, recent studies have shown that children and adolescents with type 1 diabetes exhibit a reduction in muscle strength and endurance.^9^ Additionally, research indicates that alterations in body composition, including increased body fat,^10^ further exacerbate the risk of cardiometabolic issues in this population. Despite these known effects, there is a notable scarcity of data regarding muscular fitness specifically in individuals with type 1 diabetes. A cross-sectional study involving 95 children with type 1 diabetes revealed significantly lower relative muscle power/force, influenced by the duration of the disease but not by glycated haemoglobin (HbA1c) levels.^11^ Another study involving 251 Indian children and adolescents with type 1 diabetes yielded similar results, suggesting that handgrip strength was not correlated with HbA1c levels.^12^ The link between muscular fitness and cardiometabolic health in youth with type 1 diabetes needs further exploration to inform clinical practices aimed at reducing their increased cardiovascular risk. Therefore, we aimed to investigate the relationship between handgrip strength, cardiometabolic risk (CMR) factors and body composition in these individuals both cross-sectionally and prospectively.

## Materials and methods

The present study followed the Strengthening the Reporting of Observational Studies in Epidemiology guidelines (version 4) for reporting observational studies.

### Study design, setting and participants

This study, conducted in the Autonomous Community of Navarra, Spain, was both cross-sectional and longitudinal, and involved a cohort of children and adolescents with type 1 diabetes. The participants were recruited from the Pediatric Diabetes Unit of the University Hospital of Navarra between May 2021 and February 2022. Recruitment was carried out based on the following criteria: patients aged between 6 and 18 years old, diagnosed with type 1 diabetes for more than 6 months, and willing to participate. Exclusion criteria included any comorbidity restricting participation in PA, such as CVD or severe obesity, being in the honeymoon phase (ie, all our patients met the criteria of having insulin requirements of higher than 0.5 U/kg/day and an HbA1c level higher than 6%),^13^ or a lack of sufficient understanding of the Spanish language. However, no participants were ultimately excluded from the study.

Of the 183 patients under the care of the Pediatric Diabetes Unit, 143 met the recruitment criteria. Parents or legal guardians of minors participating in the study provided written consent, while children and adolescents signed an assent form to indicate their voluntary agreement during the baseline assessment. 83 patients (58%) initially agreed to participate. At the 1 year mark, they were contacted via telephone to schedule a new evaluation, during which they could choose whether to continue. Of these, 61 patients completed the 1st-year follow-up, resulting in a dropout rate of 26.5%.

In evaluating the statistical power of our linear regression model, we used the *pwr* package in R. The model, featuring one predictor variable and three covariates, assumed an effect size of 0.5 (R²), a significance level (α) of 0.05 and a sample size of 83. The power analysis, accounting for df, yielded an estimated statistical power of approximately 0.656. This moderate power level increased confidence in our ability to identify meaningful relationships in the complex model with multiple predictors and covariates.

### Anthropometric and body composition parameters

Standing height was determined with a SECA 213 stadiometer (Hamburg, Germany). Participants were in bare feet, with heels together and touching the base of the vertical measuring column, with the back straight and with the head positioned in the Frankfurt horizontal plane.^14^ Sitting height was measured with the participant sitting on a sitting height bench (RealMet Institute, Spain), in the upright position with the feet on the floor and the head in the Frankfurt horizontal plane.^15^ Measures were recorded to the nearest 0.1 cm.

Body weight, total body fat, visceral adipose tissue (VAT) and subcutaneous adipose tissue (SAT) were measured by dual-energy X-ray absorptiometry (DXA Lunar iDXA GE Healthcare, Madison, WI), with the participant lying in supine position, with the arms slightly separated from the body and with the feet and legs hip-width apart. Appendicular skeletal muscle mass (ASM) was calculated as the sum of the muscle mass of the arms and legs. We also calculated ASM after adjusting for height squared (ASM/ht^2^).

Body mass index (BMI) was calculated by dividing the weight in kilograms by the square of height in metres. Peak height velocity (PHV), a common indicator of somatic maturity, was calculated according to Moore’s equation.^16^ Years from PHV were obtained by subtracting the age of PHV from the chronological age. The difference in years was defined as a value of maturity offset.

### Handgrip strength

A hand dynamometer with an adjustable grip (Takei Hand Grip Dynamometer, TKK-5401, Japan) was used for this test, and the grip was positioned according to the sex and hand size of each participant. The measure was carried out with the participant standing upright, facing forward, with outstretched arms slightly separated from the body, palms facing inward, and legs and feet separated hip-width apart.^17^ Each participant was instructed to grip as strong as possible and continuously for 2 s with each hand. The test was performed twice with each hand (alternating right and left sides) and results were recorded in kilograms. In case of more than 10% difference between both tests carried out with the same hand, a third measure was performed. An average of all measures was done and this was divided by the weight in kilograms in order to standardise the measures and avoid potential bias due to body weight.^17^ Patients were categorised as meeting the handgrip strength (kg/kg of body mass) according to the following thresholds associated with CMR in Spanish youth^18^: females<12 years old≥0.306 and >12 years old≥0.423; males<12 years old≥0.367 and >12 years old≥0.473. Subsequently, based on compliance at both time points, we categorised the youths into two groups: those who adhered to the recommendation at both the initial assessment and follow-up, and the remaining subjects (ie, those who did not comply at both time points).

### Cardiometabolic factors

The following data were obtained from the medical records: high-density lipoprotein cholesterol (HDL-C), low-density lipoprotein cholesterol (LDL-C), triglycerides, HbA1c (%) and fasting glucose. Medical records were also used to obtain the disease duration and type of treatment (insulin pump or multiple daily injections) of each patient. Patients with HbA1c ≤7% were categorised as meeting the glycaemic target,^5^ and those over 7% as not meeting the glycaemic goal.

Blood pressure was determined after a 10 min resting phase with the patient in supine position. The VaSera device (Fukuda Denshi, Japan) was used for this purpose according to the manufacturer’s instructions.

The triglyceride-glucose index, a measure of insulin sensitivity, was calculated according to the following equation: Ln(triglyceride (mg/dL)×fasting glucose (mg/dL)/2).^19^

Scores of each CMR factor were calculated based on their age-predicted reference values when available, as suggested by Stavnsbo *et al*.^20^ For those factors of which reference values were not available (total body fat, HbA1c, triglyceride-glucose index) specific z-scores were calculated based on the study population. A continuous CMR score was obtained by adding the following z-scores: total body fat, systolic blood pressure, diastolic blood pressure, HbA1c, LDL-C, HDL-C and triglyceride-glucose index. The z-score of HDL-C, was previously multiplied by −1 due to its inverse association with CMR.^21^ The CMR score was categorised as high if the sum was equal or over 1.

### Moderate-vigorous PA

The volume and intensity of moderate-vigorous PA (MVPA) was objectively measured with a GENEActive triaxial accelerometer (ActivInsights, Kimbolton, Cambridgeshire, UK) worn on the wrist of the non-dominant hand. Accelerometers measured at a frequency of 87.5 Hz during 9 consecutive days.^22^ Accelerometer data were extracted using the GENEActiv PC Software (V.3.3), and processed and analysed using the R-package GGIR.^23^ The following validated cut-points for MVPA were used^24^,^26^: moderate PA (for children: 191.6–695.8 mg; for adolescents: 150–500 mg) and vigorous PA (for children: >695.8 mg; for adolescents: >500 mg). In order to reduce signals related to random wrist movement, only activities for which at least 80% of 1 min time satisfied the moderate PA threshold criteria were defined as MVPA.^27^

### Statistical methods

Sample characteristics are described through summary measures (mean, SD, %). The normality of variable distributions was assessed using normality plots and the Shapiro-Wilk test. A Bonferroni adjustment was applied to maintain overall significance. Differences in variables at baseline and 1-year follow-up were analysed using either paired Student’s t-tests or Wilcoxon signed-rank tests. Dropout analysis comparing those who dropped out and continued in the first year was conducted. To address potential bias from missing data, multiple imputation methods were employed using the mice package.^28^ A total of 17 imputed datasets were generated, with each involving five iterations to ensure accuracy.^29^ Healthy convergence, imputed distribution and assumption plausibility were checked.

Since there were no differences between sexes in the categorised handgrip strength at any time point, and with the aim of increasing statistical power, we conducted the analysis combining boys and girls. We assessed differences in continuous dependent variables between participants classified with high handgrip strength and those with low grip strength both cross-sectionally and longitudinally (using baseline handgrip strength). In the former, HbA1c and CMR analyses were adjusted for PHV, type of therapy and MVPA, while body composition analyses were adjusted for PHV and MVPA. In the prospective analyses, in addition to the covariates mentioned before, each variable at baseline was considered for adjusting its corresponding variable analysis, namely HbA1c at baseline for HbA1c prospective analysis, CMR at baseline for CMR prospective analysis and so on. Additionally, we assessed differences in changes of dependent variables between participants classified with high handgrip strength at either time point or those who did not meet the cut-off. A bootstrapping method with 5000 replicates and resampling of dependent variables with replacement was used as a non-parametric method. The coefficient estimates were based on the median of the bootstrap distribution.

Finally, logistic regression analyses were conducted to assess the odds of having unhealthy HbA1c or high CMR in relation to handgrip strength (with those who did not meet the cut-off serving as the reference group), using the aforementioned adjustments.

All data analyses were performed by RStudio software (V.4.0.2-2023.12.1; RStudio; USA). Statistical significance was set up at p<0.05.

## Results

Baseline involved 83 participants with a mean age of 12.77 (±2.76) years. Of these, 37 44.58% were females and 61 of them continued with the first-year follow-up. Despite the number of dropouts, no significant differences were observed in several of the assessed parameters between those who dropped out and those who continued to participate (online supplemental table 1). Participant characteristics at baseline and 1-year follow-up are presented in table 1. 43% displayed above threshold for handgrip strength at both timepoints (ie, baseline and at 1-year follow-up). Only 35% and 36% met recommended HbA1c levels at baseline and follow-up, respectively. Finally, 83% of participants showed high CMR scores at baseline, while 75% maintained high CMR scores at follow-up.

**Table 1:** Baseline and follow-up participant characteristics of the Diactive-1 Cohort Study

	At baseline (n=83)	At 1-year follow-up (n=61)
Age, years	12.77 (2.76)	13.67 (2.74)
Sex, girls n (%)	37 (44.58)	29 (47.54)
Height, m	157.14 (16.05)	161.20 (14.19)
Weight, kg	51.57 (17.00)	55.40 (16.44)
Body mass index, kg/m²	20.39 (4.12)	20.89 (4.25)
Peak height velocity	−0.47 (1.78)	−0.12 (2.72)
Diabetes duration, years	4.84 (3.45)	5.78 (3.60)
Insulin pump, n (%)	29 (34.94)	31 (50.82)
Insulin dose, U/kg/day	0.75 (0.32)	0.73 (0.36)
Muscular fitness		
Handgrip strength, kg	21.49 (8.04)	23.14 (8.99)
Handgrip strength/weight	0.42 (0.08)	0.42 (0.09)
Handgrip strength/weight categorised*, n (%)	36 (43.37)	26 (42.62)
Physical activity		
MVPA minutes per day	38.44 (30.10)	33.09 (29.34)
Body composition		
Total body fat, %	27.82 (7.99)	27.71 (7.91)
VAT, cm³	135.30 (141.99)	137.38 (114.54)
SAT, cm³	606.64 (622.82)	603.95 (532.65)
Fat-free mass, kg	35.70 (11.81)	38.20 (11.52)
ASM, kg	16.29 (6.01)	17.56 (5.92)
ASM/ht^2^, kg/m^2^	6.38 (1.39)	6.58 (1.39)
Cardiometabolic risk factors		
HDL-C, mg/dL	62.04 (13.79)	62.21 (13.12)
LDL-C, mg/dL	97.43 (21.41)	101.54 (21.71)
Triglycerides, mg/dL	60.72 (23.09)	56.82 (20.16)
Glycated haemoglobin, %	7.42 (0.84)	7.37 (0.83)
Glycated haemoglobin >7%, n (%)	54 (65.06)	39 (63.93)
Fasting glucose, mg/dL	186.36 (72.93)	182.36 (66.97)
Systolic blood pressure, mm Hg	115.10 (11.22)	117.36 (11.99)
Diastolic blood pressure, mm Hg	68.77 (6.88)	68.70 (8.14)
CMR score, z	3.99 (3.32)	3.93 (3.33)
High CMR score†, n (%)	69 (83.13)	46 (75.41)

Triglyceride-glucose index: Ln(triglyceride (mg/dL)×fasting glucose (mg/dL)/2); CMR: cardiometabolic risk (sum of z-scores of: total body fat+systolic blood pressure+diastolic blood pressure+glycated haemoglobin+LDL-C+HDL-C+triglyceride-glucose index).

* Under 12 years: female ≥0.306, male: ≥0.367; ≥12 years: female ≥0.423, male ≥0.473 kg/kg of body mass.

† The cardiometabolic risk score was categorised as high if the sum was equal or over 1.

ASMappendicular skeletal muscle massCMRcardiometabolic riskHDL-Chigh-density lipoprotein cholesterolLDL-Clow-density lipoprotein cholesterolMVPAmoderate-vigorous physical activityMVPAmoderate-vigorous physical activitySATsubcutaneous adipose tissueVATvisceral adipose tissue

Table 2 shows cross-sectional and prospective differences between high and low handgrip strength in body composition and CMR factors. Children and adolescents classified with high levels of handgrip strength demonstrated lower levels of CMR score (3.34 (2.27 to 4.42) vs 4.48 (3.54 to 5.42), p=0.022), total body fat (23.65% (21.50 to 25.80) vs 31.02% (29.14 to 32.89), p<0.001), VAT (99.50 cm³ (55.90 to 143.09) vs 162.73 cm³ (124.73 to 200.72), p=0.035) and SAT (300.42 cm³ (116.56 to 484.27) vs 841.19 cm³ (680.96 to 1001.41), p<0.001) compared with their counterparts with low handgrip strength, assessed cross-sectionally. Prospectively, participants identified with elevated handgrip strength levels at baseline exhibited reduced levels of HbA1c (7.16% (6.85 to 7.47) vs 7.44% (7.14 to 7.74), p<0.001), CMR score (2.77 (1.78 to 3.76) vs 5.01 (4.06 to 5.97), p<0.001) and SAT (399.80 cm³ (152.96 to 646.65) vs 793.59 cm³ (551.86 to 1035.32), p=0.014) in contrast to their peers with lower handgrip strength levels 1 year later, as evidenced in table 2. Furthermore, ASM was significantly higher in the high-strength group (16.99 kg (15.66 to 18.32) vs 14.77 kg (13.35 to 16.19), p=0.030), and ASM adjusted for height squared (ASM/ht²) was also greater (6.86 kg/m² (6.40 to 7.32) vs 6.16 kg/m² (5.68 to 6.64), p=0.046). Additionally, high handgrip strength was associated with reduced odds of a high CMR score both cross-sectionally (OR=0.67, 95% CI 0.19 to 0.95, p=0.033) and longitudinally (OR=0.58, 95% CI 0.36 to 0.84, p=0.013). Imputed data analysis yielded similar prospective outcomes (online supplemental table 2).

**Table 2:** Cross-sectional and prospective differences between high and low handgrip strength in body composition and cardiometabolic risk factors

	Cross-sectional			Prospective at 1 year		
	High handgrip strengthMD (95% BCa CI)	Low handgrip strengthMD (95% BCa CI)	P value	High handgrip strength at baselineMD (95% BCa CI)	Low handgrip strength at baselineMD (95% BCa CI)	P value
Glycated haemoglobin, %	7.32 (7.07 to 7.57)	7.54 (7.25 to 7.83)	0.257	7.16 (6.85 to 7.47)	7.44 (7.14 to 7.74)	**<0.001**
Glycated haemoglobin >7%	*0.43 (0.15 to1.26*)		0.125	*0.52 (0.12 to2.20*)		0.373
CMR score, z	3.34 (2.27 to 4.42)	4.48 (3.54 to 5.42)	**0.022**	2.77 (1.78 to 3.76)	5.01 (4.06 to 5.97)	**<0.001**
High CMR score*	*0.67 (0.19 to0.95*)		**0.033**	*0.58 (0.36 to0.84*)		**0.013**
Total body fat, %	23.65 (21.50 to 25.80)	31.02 (29.14 to 32.89)	**<0.001**	28.06 (26.58 to 29.55)	27.93 (26.51 to 29.35)	0.886
VAT, cm³	99.50 (55.90 to 143.09)	162.73 (124.73 to 200.72)	**0.035**	128.70 (89.16 to 168.24)	144.32 (105.65 to 182.99)	0.541
SAT, cm³	300.42 (116.56 to 484.27)	841.19 (680.96 to 1001.41)	**<0.001**	399.80 (152.96 to 646.65)	793.59 (551.86 to 1035.32)	**0.014**
Fat-free mass, kg	36.71 (34.96 to 38.46)	34.39 (32.38 to 36.40)	0.092	37.84 (36.52 to 39.16)	37.67 (36.41 to 38.94)	0.844
ASM, kg	16.95 (16.02 to 17.88)	15.64 (15.59 to 16.70)	0.073	16.99 (15.66 to 18.32)	14.77 (13.35 to 16.19)	**0.030**
ASM/ht^2^, kg/m^2^	6.51 (6.14 to 6.88)	6.12 (5.75 to 6.50)	0.162	6.86 (6.40 to 7.32)	6.16 (5.68 to 6.64)	**0.046**

Glycated hemoglobinhaemoglobin and CMRCMR were adjusted for peak height velocity, type of therapy and moderate-vigorous physical activity. Body composition analyses were adjusted for peak height velocity and moderate-vigorous physical activity..

Triglyceride-glucose index: Ln(triglyceride (mg/dL)×fasting glucose (mg/dL)/2). The values in bold indicate significant results p<0.05, and in italics show ORs.

* The cardiometabolic risk score was classified as high if the sum was equal to or greater than 1.

ASMappendicular skeletal muscle massBCabias-corrected and acceleratedCMRcardiometabolic riskMDmean differenceSATsubcutaneous adipose tissueVATvisceral adipose tissue

**Figure 1:**
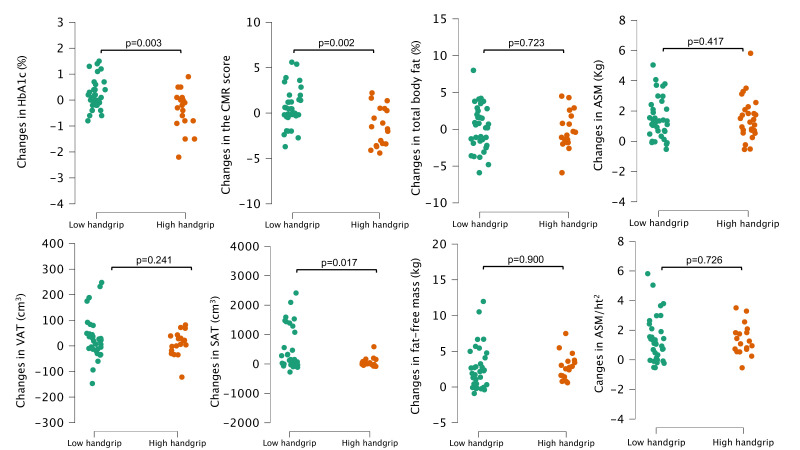
Differences in changes on cardiometabolic risk and body composition parameters in those who met the high handgrip strength criteria at both time points compared with those who have never complied or lost their compliance during the follow-up. HbA1c, glycated haemoglobin; ASM, appendicular skeletal muscle mass; CMR, cardiometabolic risk; SAT, subcutaneous adipose tissue; VAT, visceral adipose tissue.

Figure 1 shows differences in changes on cardiometabolic and body composition parameters in those who met the high handgrip strength criteria at both time points compared with those who have never complied or lose their compliance during the follow-up. Considering the compliance with the high handgrip strength cut-off points, those who met the criteria at both time points (34.4% of the sample), showed reduced levels of HbA1c (p=0.003), CMR score (p=0.002) and SAT (p=0.017), compared with those who have never complied or lost their compliance during the follow-up.

Finally, subjects classified with high handgrip strength both at baseline and at follow-up also exhibit a lower likelihood of being classified with high CMR (OR=0.241, 95% CI 0.121 to 0.947, p=0.044), but not with healthy HbA1c levels (OR=0.489, 95% CI 0.122 to 1.961, p=0.312) (data not shown).

## Discussion

This study sought to examine the association between handgrip strength and CMR, as well as body composition in youth with type 1 diabetes. Our study showed that high handgrip strength was associated with lower CMR, and less body fat compared with counterparts categorised with low handgrip strength. Additionally, our analysis revealed a longitudinal association between handgrip strength and increased ASM, suggesting enhanced muscle health and function over time.^30^

Aligning with Wallymahmed *et al*^31^ in 141 type 1 diabetes adults, handgrip strength was inversely associated with total body fat and SAT, and directly associated with fat-free mass. Possible mechanisms include muscle exerting endocrine effects via myokines, increasing lipolysis, thermogenesis and reducing fat mass.^32^ However, type 1 diabetes disturbs these pathways through hormonal dysregulation, intramyocellular lipid accumulation, protein degradation and muscle fibre structure changes.^9^ This agrees with studies linking muscular fitness to lower central adiposity in youth,^33^ possibly through enhanced muscular quality, intramyocellular lipid reductions, increased oxidative capacity and favourable muscle fibre type shifting.^34^

Despite the absence of observed cross-sectional associations, higher baseline handgrip strength was associated with lower levels of HbA1c 1 year later, as well as maintaining this level during the year of follow-up. This suggests that maintaining high strength levels could guard against prolonged hyperglycaemia over the long term. Supporting our findings, a supervised progressive resistance training regimen conducted two times per week for a minimum of 32 weeks resulted in a significant decrease in blood glucose levels, particularly HbA1C, in children with type 1 diabetes. Additionally, exercise-induced increases in adiponectin levels, which can enhance insulin sensitivity, underscore the potential benefits of resistance training for managing diabetes-related metabolic health.^8^ Similarly, a 3-month resistance training trial improved insulin sensitivity in obese adolescents.^34^ Potential mechanisms include heightened insulin sensitivity, activation of GLUT-4 transporters leading to increased glucose uptake by muscles, regulation of metabolic processes^35^ and elevation of adiponectin levels,^36^ which collectively contribute to improved metabolic health in response to resistance training in type 1 diabetes.

Regarding CMR, handgrip strength was consistently associated with lower risk scores cross-sectionally and prospectively, agreeing with studies among apparently healthy paediatric population.^37^ Our results also showed that maintaining a high level of handgrip strength was associated with a lower likelihood of being classified as having a high CMR. Previous longitudinal analyses remain conflicted, with some suggesting a protective cardiovascular effect^38^ while others show no impact.^39^ Possible pathways include improved endothelial function, anti-inflammatory, apoptotic and insulin sensitivity effects.^40^ Our findings support the use of type 1 diabetes youth CMR thresholds,^18^ offering a valuable clinical tool.

In conclusion, our results suggest that maintaining handgrip strength may be associated with favourable cardiovascular and body composition profiles in youth with type 1 diabetes. Therefore, handgrip strength, as a surrogate marker, presents itself as a promising and cost-effective metric for predicting future health outcomes. Its utility extends beyond mere assessment, warranting careful integration into clinical practice as both a screening tool and therapeutic target. Incorporating handgrip strength assessment into routine clinical evaluations could provide clinicians with valuable insights into CVD risk.

### Strengths and limitations

Our primary strength was the combined cross-sectional and longitudinal analysis. Additionally, dual-energy X-ray absorptiometry enhances body composition precision over alternative techniques.^41^ However, some limitations warrant consideration. First, handgrip strength may insufficiently represent overall muscle strength compared with additional lower- and upper-body assessments. Nonetheless, handgrip strength validly predicts total strength in healthy youth.^42^ Second, inconsistencies defining the most suitable CMR factors restrict result comparisons. Finally, our localised sample hinders generalisation.

## supplementary material

10.1136/bmjsem-2024-002177 online supplemental file 1

## Data Availability

Data are available upon reasonable request.
