# Supplementary material for: Handgrip strength, cardiometabolic risk and body composition in youth with type 1 diabetes: the Diactive-1 Cohort Study
Source: BMJ Open Sport Exerc Med. 2024 Dec 4;10(4):e002177. doi: 10.1136/bmjsem-2024-002177 (PMC11624725; doi:10.1136/bmjsem-2024-002177)
Supplement: online supplemental file 1 [file bmjsem-10-4-s001.pdf]

Supplementary Material

Supplementary Table 1. Differences between participants who discontinued the study and those who remained for the first-year follow-up.

	Outs	Followed	p
Age, years	13,05 (2,92)	12,67 (2,73)	0.590
Peak height velocity	-0.30 (2,10)	-0.27 (1,90)	0.948
Diabetes duration, years	5.08 (3.04)	4.75 (3.60)	0.702
Physical fitness parameters			
Handgrip strength/weight	0.40 (0.09)	0.42 (0.07)	0.287
Physical activity parameters			
MVPA min/d	38.42 (21.95)	38.45 (32.60)	0.997
Cardiometabolic risk score and glycated hemoglobin			
Glycated hemoglobin, %	7.68 (0.69)	7.32 (0.88)	0.084
CMR Score, z	4.98 (3.39)	3.63 (3.25)	0.103
Total body fat, %	28.45 (9.19)	27.60 (7.58)	0.672
Free-fat mass, kg	68.89 (8.59)	69.70 (7.19)	0.667
ASM, kg	16.30 (5.99)	16.31 (6.12)	0.723
ASM/ht^2, kg/m^2	6.38 (1.56)	6.40 (1.41)	0.546

Abbreviations: ASM: Appendicular Skeletal Muscle Mass; CMR: cardiometabolic risk; min/d: minutes per day; MVPA: moderate-vigorous physical activity.

Supplementary Table 2. Prospective differences between high and low handgrip strength at baseline in body composition and cardiometabolic risk factors at 1-year follow-up with imputed data.

	High handgrip (95% CI)	Low handgrip (95% CI)	p
Glycated hemoglobin, %	7.37 (7.14 to 7.81)	7.74 (7.45 to 8.03)	**0.042**
Glycated hemoglobin >7%	*0.59 (0.17 to 1.99)*		0.394
CMR Score, z	3.83 (3.02 to 4.63)	4.94 (4.24 to 5.64)	**0.045**
High CMR Score §	*0.45 (0.19 to 0.92)*		**0.040**
Total body fat, %	28.48 (27.43 to 29.54)	27.37 (26.46 to 28.28)	0.137
VAT, cm^3	142.99 (113.89 to 172.09)	167.56 (142.25 to 192.87)	0.219
SAT, cm^3	657.52 (557.76 to 757.28)	666.94 (580.97 to 752.92)	0.893
Fat-free mass, kg	38.62 (37.62 to 39.63)	38.84 (37.97 to 39.72)	0.747
ASM, kg	17.00 (15.31 to 18.67)	14.99 (14.35 to 16.21)	**0.045**
ASM/ht^2, kg/m^2	6.86 (6.11 to 7.45)	6.21 (5.18 to 6.64)	**0.041**

Glycated hemoglobin analyses adjusted for glycated hemoglobin at baseline, peak height velocity, type of therapy and moderate-vigorous physical activity. CMR score analyses adjusted for CMR score at baseline, peak height velocity, type of therapy and moderate-vigorous physical activity. Body composition analyses adjusted for each body composition variable at baseline, peak height velocity and moderate-vigorous physical activity. Triglyceride-glucose index analyses adjusted for triglyceride-glucose index at baseline, peak height velocity, type of therapy and moderate-vigorous physical activity. The values in bold indicate significant results $p < 0.05$, and in italics show odds ratios.

Abbreviations: ASM: Appendicular Skeletal Muscle Mass; CMR: cardiometabolic risk; SAT: subcutaneous adipose tissue; VAT: visceral adipose tissue. § Cardiometabolic risk: z score ≥1.0. Triglyceride-glucose index: Ln(Triglyceride (mg/dL) x fasting glucose (mg/dL)/2).
